# Microglia mechanics: immune activation alters traction forces and durotaxis

**DOI:** 10.3389/fncel.2015.00363

**Published:** 2015-09-23

**Authors:** Lars Bollmann, David E. Koser, Rajesh Shahapure, Hélène O. B. Gautier, Gerhard A. Holzapfel, Giuliano Scarcelli, Malte C. Gather, Elke Ulbricht, Kristian Franze

**Affiliations:** ^1^Department of Physiology, Development and Neuroscience, University of CambridgeCambridge, UK; ^2^Faculty of Computer Science and Biomedical Engineering, Institute of Biomechanics, Graz University of TechnologyGraz, Austria; ^3^Department of Physics, Institute for Theoretical Physics, University of CologneCologne, Germany; ^4^Fischell Department of Bioengineering, University of MarylandCollege Park, MD, USA; ^5^Scottish Universities Physics Alliance (SUPA), School of Physics and Astronomy, University of St AndrewsSt Andrews, UK

**Keywords:** migration, mechanotaxis, foreign body reaction, random walk, LPS, CNS, gliosis, biased random walk

## Abstract

Microglial cells are key players in the primary immune response of the central nervous system. They are highly active and motile cells that chemically and mechanically interact with their environment. While the impact of chemical signaling on microglia function has been studied in much detail, the current understanding of mechanical signaling is very limited. When cultured on compliant substrates, primary microglial cells adapted their spread area, morphology, and actin cytoskeleton to the stiffness of their environment. Traction force microscopy revealed that forces exerted by microglia increase with substrate stiffness until reaching a plateau at a shear modulus of ~5 kPa. When cultured on substrates incorporating stiffness gradients, microglia preferentially migrated toward stiffer regions, a process termed durotaxis. Lipopolysaccharide-induced immune-activation of microglia led to changes in traction forces, increased migration velocities and an amplification of durotaxis. We finally developed a mathematical model connecting traction forces with the durotactic behavior of migrating microglial cells. Our results demonstrate that microglia are susceptible to mechanical signals, which could be important during central nervous system development and pathologies. Stiffness gradients in tissue surrounding neural implants such as electrodes, for example, could mechanically attract microglial cells, thus facilitating foreign body reactions detrimental to electrode functioning.

## Introduction

The central nervous system (CNS) is separated from the systemic immune system by the blood–brain barrier, which protects the tissue from most infections. When pathogens cross the blood-brain-barrier, after brain lesions, or in any other CNS dysfunction, microglial cells form the first line of immune defense. After detection of a pathological signal, microglia become activated and migrate toward the stimulus, where they release a large number of signaling molecules, proliferate, and phagocytose cells and debris (Kettenmann et al., 2011).

Chemical interactions of microglial cells with their environment have been extensively studied in the past. However, in order to migrate, microglia need to exert forces on their environment. While they exert forces on the surrounding tissue, they probe its mechanical properties (Franze et al., 2013). Yet, forces exerted by microglial cells have not been measured, and their mechanical interactions with their environment are currently poorly understood.

Glial cells respond to the stiffness of their environment in different ways. Astrocytes, for example, increase their spread area, overall morphological complexity, and F-actin organization on stiffer growth substrates (Georges et al., 2006; Moshayedi et al., 2010). Similarly, oligodendrocyte progenitor cells increase their area and complexity on stiffer substrates, but show an optimum in survival and proliferation at intermediate values of CNS tissue stiffness (Jagielska et al., 2012). Morphologies of microglial cells also become more complex on stiffer substrates, and similarly to astrocytes, they upregulate inflammatory mediators when exposed to an environment with increased stiffness (Moshayedi et al., 2014).

Stiff neural implants, for example, were shown to activate and attract microglia and astrocytes, while soft implants that were mechanically matched to the surrounding CNS tissue diminished this foreign body reaction *in vivo* (Moshayedi et al., 2014). It was suggested that the large stiffness of a common neural implant, such as an electrode, triggers microglia migration toward that foreign body (Franze et al., 2013), in a similar fashion as some cell types migrate from softer to stiffer matrices in a process termed durotaxis (Lo et al., 2000). However, while substrate stiffness was shown to regulate glial cell migration (Mori et al., 2013; Kim et al., 2014), direct experimental proof for an impact of the mechanical properties of the surrounding on microglia migration is currently missing.

CNS tissue is mechanically heterogeneous at a length scale relevant to individual cells (Elkin et al., 2007; Christ et al., 2010; Franze et al., 2011; Iwashita et al., 2014; Koser et al., 2015). Furthermore, its mechanical properties may alter with age (Sack et al., 2011; Arani et al., 2015) and in pathological conditions (Murphy et al., 2011; Riek et al., 2012; Schregel et al., 2012; Streitberger et al., 2012; Chauvet et al., 2015). Thus, microglia are exposed to varying mechanical signals on their way to sites of damage. To test if these signals may impact the interactions of microglial cells with their environment, we measured traction forces exerted by microglia as a function of substrate stiffness. We furthermore investigated their migratory behavior on substrates with stiffness gradients, and developed a model to predict microglia migration based on their traction forces.

## Materials and methods

All chemicals were purchased from Sigma-Aldrich (Sigma-Aldrich Company Ltd., Gillingham, UK), unless otherwise stated.

### Polyacrylamide substrates

To obtain deformable cell culture substrates of varying stiffness or incorporated stiffness gradients, modified protocols of Grevesse et al. (2013) and Moshayedi et al. (2010) were used. Substrates were made of polyacrylamide (PAA), which is a transparent, homogeneous, isotropic, and linearly elastic material. PAA gels were polymerized on imaging dishes (μ-Dish, Ibidi, Germany) for traction force microscopy and on coverslips otherwise. Surfaces of the imaging dishes or coverslips were cleaned with 70% ethanol and made hydrophilic with 0.1% sodium hydroxide (NaOH). (3-Aminopropyl) trimethoxysilane (APTMS) was applied for a duration of 3 min to the NAOH-treated surface. Subsequently, it was washed and covered with 0.5% glutaraldehyde for 30 min. PAA stock solutions for homogeneous substrates were made of 500 μl 40% acrylamide (AA), 65 μl 100% hydroxy-acrylamide (OH-AA) and 250 μl 2% bis-acrylamide (Bis-AA, Fisher scientific, UK). PAA premixes for gradient substrates were made according to Moshayedi et al. (2010).

#### Preparation of PAA substrates for traction force microscopy

Fluorescent nanoparticles (FluoSpheres carboxylate, 0.2 μm, crimson, Life Technologies, UK) were added to the PAA premixes, which were then placed in an ultrasonic bath for 30 s to separate the beads. Subsequently, premixes were degassed for 10 min. Adding 1.5 μL N,N,N′,N′-tetramethyl-ethylenediamine (TEMED) and 5 μL of a 10% ammonium persulfate solution (APS) initiated the cross-linking of the gels. Immediately thereafter, 8 μl of the solution were pipetted on the imaging dish. A coverslip that had been cleaned and made hydrophobic with RainX (Kraco Car Care International Ltd., UK) was lowered onto the drop to create a gel layer of even thickness. The imaging dish was then inverted to ensure that beads settled close to the gel surface. Once the gel had polymerized, the surface was covered with PBS and the coverslip was removed. The gels were subsequently washed and sterilized under UV light for ~15 min. To promote cell adhesion, gel surfaces were treated with 100 μg/ml poly-D-lysine (PDL) for 2 h.

#### Preparation of stiffness gradient substrates

Two Parafilm-covered microscope slides were used to enclose a glutaraldehyde-treated 22 × 22 mm^2^ coverslip and a 22 × 40 mm^2^ coverslip cleaned and made hydrophobic with RainX. The treated sides were facing each other and separated by a U-shaped spacer made of Parafilm. Bulldog clips were used to hold the chamber together (Koser et al., under review).

PAA premixes for substrates with shear moduli of *G*′ ~ 100 Pa and *G*′ ~ 10 kPa were prepared as described in Moshayedi et al. (2010). In the 10 kPa premix, 5 μl of PBS were substituted by 5 μL of 1% (w/v) fluorescein O,O′-dimethacrylate diluted in DMSO. The premix solutions were vortexed and then desiccated for 10 min. Polymerization was initiated as described above. The bottom half of the chamber was then filled with the premix of the stiff substrate (*G*′ ~ 10 kPa), the upper half subsequently with the premix for the soft substrate (*G*′ ~ 100 Pa). Diffusion led to overlapping linear gradients in stiffness and fluorescence intensity (Supplementary Figure 1 and Koser et al., under review). Therefore, the fluorescence intensity could be used as readout for the local substrate stiffness. Once the gel had polymerized, the chamber was submerged in PBS and the coverslips separated. The gel was sterilized under a UV lamp for 1 h and washed three times in filter-sterilized PBS. The gel was then incubated over night at room temperature in a 1:99 Cell-Tak™ (BD Biosciences)—PBS solution (Koch et al., 2012), and subsequently treated with a 1:10 poly-L-lysine (PLL)–PBS solution for 2 h.

### Microglial cell cultures

All animal experiments of this study were conducted in accordance with the UK Animals (Scientific Procedures) Act (1986). Neonatal P0-P2 Sprague Dawley rats (for TFM, actin morphology and cell size experiments) or P0–P5 mice (C57BL/6; for migration assays) were decapitated and their cortices dissected from the exposed brains, or whole brains used, respectively. Microglia were obtained following Giulian and Baker (1986) and Mccarthy and De Vellis (1980). The rat cortices were cut into small pieces, minced and incubated in a cell dissociation solution containing papaya proteinase 1 (Papain) at 37°C for 1 h. To stop papain digestion, an Ovomuccoid (Trypsin inhibitor) solution was added. Mice brains were minced mechanically and filtered through cell strainers. The cell suspension was centrifuged at 1000 rpm for 8 min. The resulting pellet of cells was suspended in glia medium [DMEM (Gibco, Life Technologies Ltd., UK) + 3.8 mM L-Glutamine + 10% fetal bovine serum (FBS) (Gibco) + 1% Penicillin Streptomycin (PenStrep) (Gibco); as described by (Moshayedi et al., 2010)] and incubated in PDL or PLL-coated T-75 flasks. The mixed glial cultures were kept at 37°C in a 5% CO_2_–incubator and the medium was changed after 12 h and thereafter every 2–3 days and. After reaching confluence, flasks were shaken overnight at about 200 rpm using an orbital shaker to promote the detachment of microglia and oligodendrocyte precursor cells (OPCs). The supernatant was then removed, leaving T-75 flasks with predominantly astrocytes. Separation of microglia and OPCs was achieved by pouring the suspension into an untreated plastic dish and incubating it for 30 min at 37°C. Microglia attached to plastic quickly and the medium containing OPCs was removed and replaced by fresh culturing medium. To shake off the microglial cells, either the plastic dish was placed on ice and shaken at 400 rpm on an orbital shaker for about 30 min, or the culture medium was replaced by 10 mL of a 9:1 HBSS:trypsin solution and incubated for 5 min. 10 mL of FBS were added to stop the trypsin activation. In both cases cell suspension was then transferred to a 15 ml tube and centrifuged at 500–1000 rpm for 1–7 min. Subsequent to removing the supernatant, the cell pellet was resuspended using glia medium or Leibovitz medium (PAA, Hamshire, UK) containing 2.05 mM L-Glutamine. The fraction of microglial cells in the final cell culture was found to be ≥95%.

### Cell staining and fluorescence imaging

Microglial cells were cultured on single stiffness PAA substrates and glass for 12 h and fixed. Before culturing, the glass was cleaned with 70% ethanol and treated with 100 μg/ml poly-D-lysine (PDL) for 2 h. In order to label F-actin, cells were washed with PBS and then fixed with 4% PFA in PBS for 10 min, permeabilized in 0.1% Triton PBS for 5 min, and then washed with PBS four times for 15 min. Cells were incubated with Alexa Fluor488 phalloidin (Life Technologies, UK) at 2 units in 300 μl per gel for 1 h. Nuclei were stained using 1 μg/mL DAPI followed by three washes in PBS for 15 min. Cover slips with gels and stained cells were then mounted onto imaging slides using Fluoromount G (SouthernBiotech, USA). For fluorescence imaging, an upright microscope (Eclipse Ni, Nikon, Japan) with a precentered fiber illuminator (intensilight C-HGFI, Nikon, Japan) was used. Images were acquired using a high sensitivity camera (iXON3, Andor Technology Ltd, UK), a 60X water immersion objective (*NA* = 1, Nikon, Japan) and NIS-Elements software (Nikon, Japan). A subset of images was recorded using an inverted Leica DMI3000 microscope (63X oil immersion objective, *NA* = 1.4) with a CCD camera (dfc 340fx).

### Time lapse imaging for TFM

The cell suspensions were seeded onto the PAA gels and kept in a 5% CO_2_- incubator for 10 min to allow for cell adhesion. An additional 2 ml of glia medium per imaging dish was then carefully added to ensure cell survival. The cells were kept at 37°C until imaging.

The imaging setup consisted of an inverted microscope (Axio Observer.A1, Carl Zeiss Ltd., UK), an EMCCD camera (iXON3, Andor Technology Ltd, UK), a HXP 200C illuminator (Zeiss, Germany) and 40X water immersion objective (*NA* = 1.1, Zeiss, Germany). A petri dish heater was used to keep the sample at ~37°C (JPK Instruments AG, Germany). Images were acquired using ANDOR Solis software. Fluorescence images of beads were taken every 30 s. After 10 min, lipopolysaccharide was added to the medium (LPS, Gibco) resulting in a final concentration of 1–2 μg/ml. After 5 min incubation time, imaging was continued for another 10 min. After the acquisition of images, Trypsin (Gibco) was added to the culture medium to detach cells from the gel. Reference images of fluorescent beads at the “relaxed” state were taken ~10 min thereafter. We performed two independent traction force experiments on four substrates of varying stiffness (*G*′ = 100 Pa, 300 Pa, 1 kPa, 10 kPa; Table 1).

**Table 1:** **Components of PAA substrates for traction force microscopy**.

***G*′ (Pa)**	**100% PBS (μL)**	**PAA stock solution (μL)**	**Beads (μL)**
~100	437	53	10
~300	432	58	10
~1000	415	75	10
~10000	340	150	10

### Time lapse measurements for cell tracking

To avoid drifting of the coverslip during the experiment, the coverslip with the stiffness gradient substrate was glued to the bottom of a petri dish. Approximately 50,000 cells in 2 ml Leibovitz medium were seeded on the gel. The petri dish was placed inside a petri dish heater (JPK Instruments AG, Germany) set to 37°C on an inverted microscope (Axio Observer.A1, Carl Zeiss Ltd., UK) using a 10x objective (EC Plan-Neofluar 10x/0.30 Ph1, Carl Zeiss Ltd., UK) and a CCD camera (1/3″ CCD fire wire color camera, The Imaging Source, UK). Images were acquired using SPM 4.0 Software (JPK Instruments AG, Germany). As the fluorescence intensity correlated linearly with the substrate stiffness (Supplementary Figure 1 and Koser et al., under review), fluorescence intensity was employed as a readout for the local substrate stiffness. Therefore, after the cells had settled, the region of interest was selected based on the fluorescence signal of the gradient and a sufficient number of cells. Pictures of the gradient gel's fluorescence were taken to calculate the absolute stiffness and the steepness of the stiffness gradient of the gel. Subsequently, time-lapse sequences of microglial cells were recorded at 1 image per minute for at least 3 h. 4 independent experiments were performed in this way. In 2 of these experiments, LPS was added in a concentration of (1–2) μg/mL after 1–4 h, and time-lapse data were acquired separately.

### Data analysis

#### Traction stresses

For the calculation of traction stresses, a modified version of the code by Koch et al. (2012) was used in MATLAB (MathWorks Inc., MA, USA). The code is based on the method of Sabass et al. (2008) and Betz et al. (2011). In order to observe traction stresses of single cells, a region of interest (ROI) was identified for each cell. To calculate the discrete deformation field for the ROI with respect to the reference image, a 2D cross-correlation algorithm was applied. The continuous deformation field was obtained by applying Gauss interpolation to the resulting discrete deformation field of the ROI. The inverse Boussinesq Green function was used in the Fourier space to calculate the traction stresses on the substrate surface from the continuous deformation field. The peak traction stress is defined by the largest value of the resulting traction stress map. The average stress is computed as the mean of all stresses that are greater than a threshold value. The threshold value was defined as 30% of the present peak traction stress value (Koch et al., 2012). Hence, one peak and one average stress value was obtained for each cell at each time step. The medians of the average and peak stresses recorded over 10 min (corresponding to 20 data points per cell) were statistically compared in case of control cells, and the median stresses recorded over 5 min (corresponding to 10 data points per cell) were compared for treated cells after incubation in LPS for 10 min.

To feed stress distributions underneath the cells in our model, we discretized the traction stress map using a spatial resolution of ~3 μm. Contours of cells were obtained from phase contrast images. Stress distributions were calculated for the first time step.

#### Cell tracking

To track cells we used an automatic custom-written tracking software based on the software of Graham Milne (PhD Thesis, University of St. Andrews, 2007). In total, 267 cells in standard medium and 128 cells in a medium with LPS were tracked. The starting position of each cell was set to the origin of the coordinate system, and the y-axis of the coordinate system was aligned with the direction of the gradient (with the positive y-direction pointing toward the stiffer side of the gradient). The mean stiffness of the gradient substrate at which the cells were tracked was ~5 kPa, the mean steepness of the stiffness gradient ~8.35 Pa/μm.

##### Selection process of actively moving cells

Cells that are not actively migrating include two subpopulations: adhered, non-motile cells and non-adhered, diffusing cells. Mean squared displacements (MSDs) of both subpopulations will be smaller or equal the MSD of a freely diffusing particle of comparable size, while we expect the MSD of actively moving cells to be larger. We thus calculated the MSD of each tracked cell and used the following inequality as a selection criterion for actively moving cells:

(1) $$MSD>\frac{k_{B}T}{3\pi \eta r}t,$$

where *k*_*B*_ is the Boltzmann constant, *T* = 310 K the absolute temperature, η the viscosity of the medium, *r* the radius of the particle and *t* the time. We obtained the average radius of the cells by measuring the diameter of 10 randomly selected cells (*r* ≈ 8 μm). Furthermore, we assumed the viscosity of the medium to be η≈1 mPa s. The used cutoff in Equation (1) is about that of the Brownian motion of a freely diffusing particle of the same size. Due to this criterion, the majority of cells (~83%) was rejected; thus, the results shown represent only a subpopulation of microglia (38 cells in the control condition and 22 LPS-treated cells).

##### Directness

The directness *D* (also known as the straightness index or the net to gross displacement ratio) is defined by

(2) $$D=\langle \frac{d_{e}}{d_{t}}\rangle ,$$

with the Euclidean distance (the shortest linear distance between the start and endpoints of a path, also called the net displacement) *d*_*e*_ and the total travel distance (or contour length) *d*_*t*_ of each cell (Codling et al., 2008), while 〈〉 represents the mean.

##### Contour and euclidean velocity

The contour velocity *v*_*c*_ is defined by

(3) $$v_{c}=\frac{\sum_{i=2}^{N}\left|{\vec{p}}_{i}-{\vec{p}}_{i-1}\right|}{t},$$

with the position ${\vec{p}}_{i}$ of the cell on the *i*th frame, the total frame number *N* and the imaging time *t*. The Euclidean velocity vector ${\vec{v}}_{e}$ and its components *v*_*e,x*_ and *v*_*e,y*_ were calculated by

(4) $${\vec{v}}_{e}=\left(\begin{array}{c} v_{e,x}\\ v_{e,y} \end{array}\right)=\frac{1}{t}\left({\vec{p}}_{s}-{\vec{p}}_{e}\right),$$

with the start position ${\vec{p}}_{s}$ and end position ${\vec{p}}_{e}$ of the cell.

##### Turning and direction angles

The turning angle φ is defined for the third component of the vector product $\left({\vec{p}}_{i}-{\vec{p}}_{i-1}\right)\times \left({\vec{p}}_{i-1}-{\vec{p}}_{i-2}\right)$ being ≥ 0 by

(5a) $$\varphi =\;\cos^{-1}\;\frac{\left({\vec{p}}_{i}-{\vec{p}}_{i-1}\right)\;\cdot \;\left({\vec{p}}_{i-1}-{\vec{p}}_{i-2}\right)}{\left|{\vec{p}}_{i}-{\vec{p}}_{i-1}\right|\left|{\vec{p}}_{i-1}-{\vec{p}}_{i-2}\right|},$$

and for the third component of $\left({\vec{p}}_{i}-{\vec{p}}_{i-1}\right)\times \left({\vec{p}}_{i-1}-{\vec{p}}_{i-2}\right)$ being < 0 by

(5b) $$\varphi =2\pi -\cos^{-1}\;\frac{\left({\vec{p}}_{i}-{\vec{p}}_{i-1}\right)\;\cdot \;\left({\vec{p}}_{i-1}-{\vec{p}}_{i-2}\right)}{\left|{\vec{p}}_{i}-{\vec{p}}_{i-1}\right|\left|{\vec{p}}_{i-1}-{\vec{p}}_{i-2}\right|},$$

with the position ${\vec{p}}_{i}$ of the cell on the *i*th frame. The direction angle θ is defined for the third component of $\left({\vec{p}}_{i}-{\vec{p}}_{i-1}\right)\times \vec{d}$ being ≥ 0 by

(6a) $$\begin{array}{c} \theta =\cos^{-1}\frac{\left({\vec{p}}_{i}-{\vec{p}}_{i-1}\right)\;\cdot \;\vec{d}}{\left|{\vec{p}}_{i}-{\vec{p}}_{i-1}\right|\;\left|\vec{d}\right|}, \end{array}$$

and for the third component of $\left({\vec{p}}_{i}-{\vec{p}}_{i-1}\right)\times \vec{d}$ being < 0 by

(6b) $$\begin{array}{l} \begin{array}{c} \theta =2\pi -\cos^{-1}\frac{\left({\overset{⇀}{p}}_{i}-{\overset{⇀}{p}}_{i-1}\right)\cdot \overset{⇀}{d}}{\left|{\overset{⇀}{p}}_{i}-{\overset{⇀}{p}}_{i-1}|\;|\overset{⇀}{d}\right|}, \end{array} \end{array}$$

where $\vec{d}$ is the direction vector of the gradient (pointing along the gradient and toward the stiffer side of the gradient).

We used the turning angle φ to calculate the probability of a cell to move forward, backwards, left, and right, and the direction angle θ for the probability of a cell to move along the gradient, perpendicular to it, to the stiff side, and to the soft side. A cell was defined as moving forward if φ was between −45° (315°) and 45° (backwards: 135° and 225°; left: 45°and 135°; right: 225° and 315°). A cell was defined to move along the gradient, if θ was between −45° and 45° or between 135° and 255° (perpendicular to the gradient: 45°and 135° or 225° and 315°; to the stiff side: −45° and 45°; to the soft side: 135° and 255°).

##### Correlation index of movement

The correlation index of movement (*CI*) is defined by (Codling et al., 2008)

(7a) $$CI=\sqrt{{\left(\int_{-\pi }^{\pi }\cos\varphi p(\varphi )d\varphi \right)}^{2}+{\left(\int_{-\pi }^{\pi }\sin\varphi p(\varphi )d\varphi \right)}^{2}},$$

where φ is the angle between two movement steps (i.e., the turning angle) and *p(*θ*)* is its probability density function. For our analysis we discretize the probability density function p^*^(φ*)* by binning the data into π/9 wide parts φ^*^. Therefore, *CI* is calculated by

(7b) $$CI=\sqrt{{\left(\sum_{\varphi \in \varphi^{*}}\cos\varphi p^{*}(\varphi )\right)}^{2}+{\left(\sum_{\varphi \in \varphi^{*}}\sin\varphi p^{*}(\varphi )\right)}^{2}},$$

where *CI* ∈ [0,1]. A value close to 0 suggests an uncorrelated movement and a value close to 1 a highly correlated movement.

##### Bias index of movement

The bias index of movement (*BI*) is mathematically similar to the *CI*. Instead of the turning angle φ, the direction of cells relative to the stiffness gradient θ is taken into account. The *BI* can be calculated by

(8) $$BI=\sqrt{{\left(\sum_{\theta \in \theta^{*}}\cos\theta p^{*}(\theta )\right)}^{2}+{\left(\sum_{\theta \in \theta^{*}}\sin\theta p^{*}(\theta )\right)}^{2}},$$

where *BI* ∈ [0,1]. A value close to 0 suggests an unbiased movement and a value close to 1 a highly biased movement. It has to be noted that a high *BI* can be caused by a correlated random walk; in this case *CI* would be high as well.

#### Statistical tests

Data were tested for normal distribution by the Kolmogorov-Smirnov or Lilliefors tests. When two groups were compared, significance was tested either with Student's *t*-test (in case of normal distribution) or the Mann-Whitney *U*-test (in case of non-normal distribution). If more than two groups were compared either a One-Way or Two-Way ANOVA was used in case all groups were normally distributed, or a Kruskal-Wallis ANOVA otherwise. Two-tailed tests were used throughout the analysis. In the text we mention either the mean ± SEM or the median.

## Results

### Cell morphology changes with substrate stiffness

**Figure 1:**
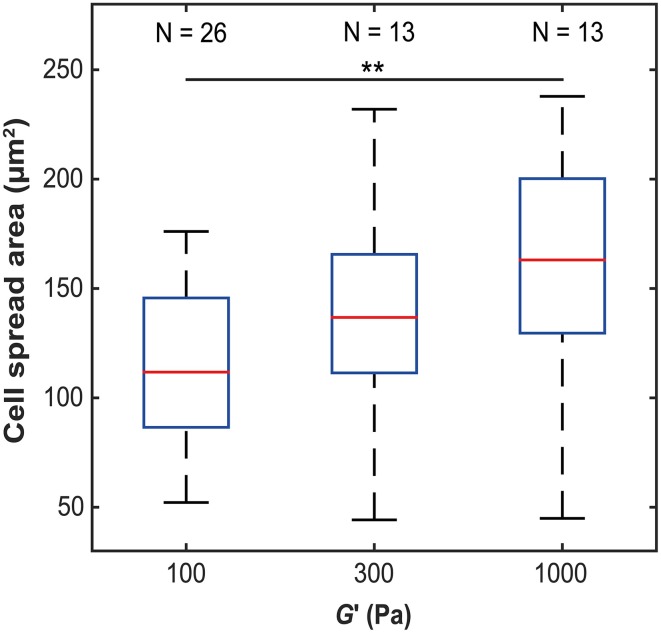
**Microglia spread area as a function of substrate stiffness**. The spread area of microglial cells significantly increased with substrate stiffness (*p* < 0.05, Kruskal-Wallis ANOVA; for *G*′ ~ 100 Pa vs. 1 kPa, *p* < 0.01, Mann-Whitney *U*-test). ^**^p < 0.01.

To investigate how microglia mechanically interact with their environment, we first investigated how their morphology changes with substrate stiffness. Primary microglial cells were cultured on soft, elastic substrates made of polyacrylamide with shear moduli *G*′ of 100, 300, and 1000 Pa, spanning the range of reported neural tissue stiffness (Franze et al., 2013). The spread area of primary microglial cells significantly increased with substrate stiffness (*p* < 0.05, Kruskal-Wallis ANOVA) (Figure 1). The median cell spread area increased from 112 μm^2^ on soft substrates (*G*′ ~ 100 Pa), to 163 μm^2^ on stiff substrates (*G*′ ~ 1000 Pa) (*p* < 0.01, Mann-Whitney *U*-test).

**Figure 2:**
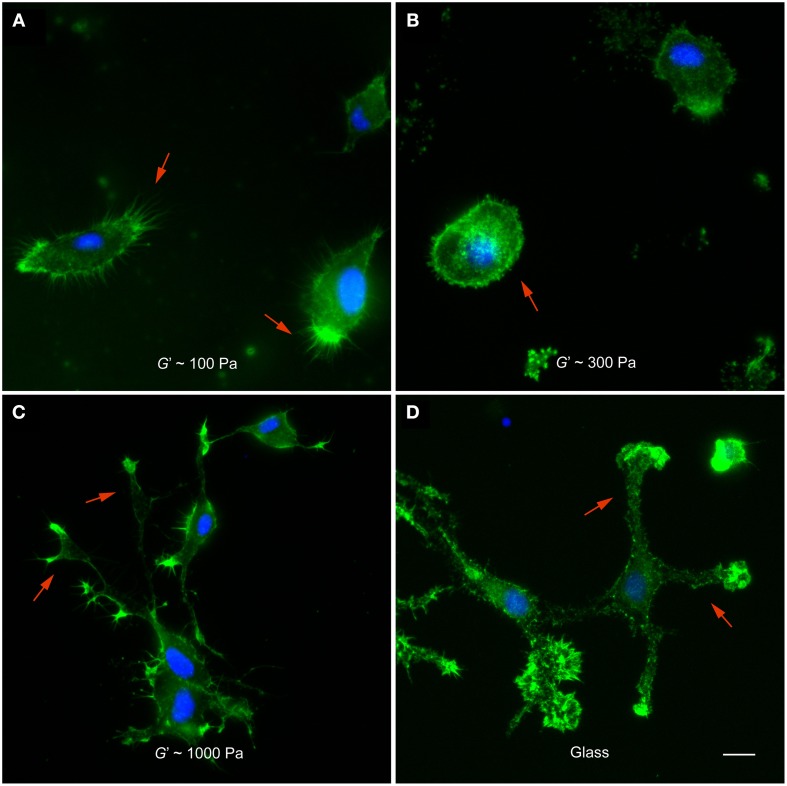
**Microglia morphology depends on substrate stiffness**. **(A–D)** Representative fluorescence images of microglial cells on substrates of different stiffness. F-actin appears in green, nuclei in blue. **(A)** On substrates of *G*′ ~ 100 Pa, microglial cells showed many filopodia-like processes (red arrows). **(B)** On stiffer substrates (*G*′ ~ 300 Pa), they showed amoeboid morphologies (red arrow) with significantly fewer processes (*p* < 0.05, Mann-Whitney *U*-test). **(C,D)** On 1000 Pa substrates and on glass, microglial cells had complex morphologies with lamellipodia-like structures at the tips of long processes (red arrows). Scale bar: 10 μm.

After 12 h *in vitro*, microglia cultured on 100 Pa substrates showed many actin-rich filopodia-like processes (44 ± 3, average ± SEM, *n* = 14) (Figure 2A). Microglial cells on substrates of *G*′~300 Pa appeared in a more round or amoeboid shape (Figure 2B), with fewer processes than on softer substrates (24 ± 6, *n* = 5; *p* < 0.05, Mann-Whitney *U*-test). Furthermore, F-actin was distributed more homogeneously throughout the cells. The shape of microglia changed drastically when cultured on 1 kPa substrates or on glass (Figures 2C,D). Cells possessed more complex morphologies with long, distinct processes and lamellipodia-like structures at their tips.

### Microglia traction stresses increase with substrate stiffness

**Figure 3:**
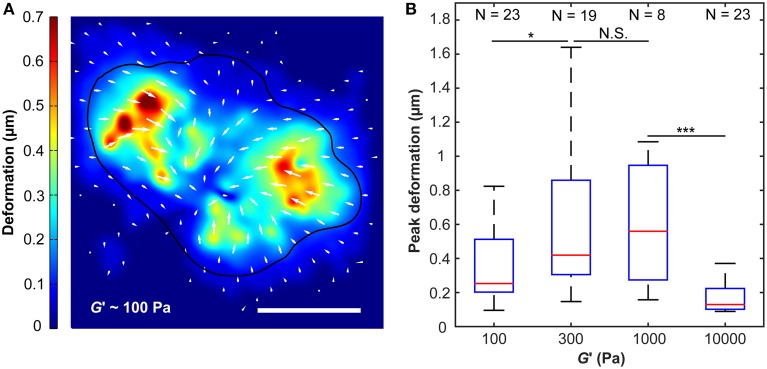
**Substrate deformations as a function of substrate stiffness**. **(A)** Substrate deformation field of one representative cell grown on a ~100 Pa substrate (cell outline in black). Deformation directions are indicated by white arrows, absolute deformations are shown by false colors. Scale bar: 10 μm. **(B)** Peak deformations reached a maximum on substrates of around *G*′ ~300–1000 Pa. N, number of analyzed cells. ^*^p < 0.05; ^***^p < 0.001.

Changes in cell spread area and in the F-actin cytoskeleton are often accompanied by changes in cellular forces (Tolic-Norrelykke and Wang, 2005; Califano and Reinhart-King, 2010; Fournier et al., 2010; Stricker et al., 2010). To measure microglial traction forces, we embedded fluorescent nanoparticles in compliant substrates, which allowed tracking substrate deformations due to forces exerted by cells (Figure 3).

Peak substrate deformations changed with substrate stiffness (*p* < 10^−6^, Kruskal-Wallis ANOVA). While maximum deformations were significantly smaller on substrates of *G*′ ~ 100 Pa compared to those of 300 Pa (*p* < 0.05, Mann-Whitney *U*-test), there was no notable difference between 300 Pa and 1000 Pa substrates. A significant decline in peak substrate deformations was observed for 10 kPa substrates if compared to 1000 Pa substrates (*p* < 10^−3^) (Figure 3).

**Figure 4:**
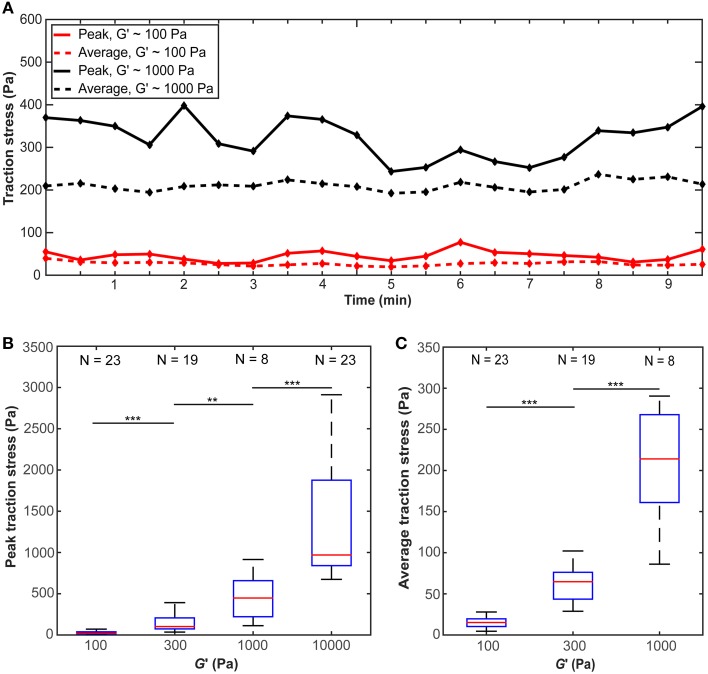
**Microglial cells exert larger forces on stiffer substrates**. **(A)** Average and peak traction stress as a function of time for two representative cells on gels of *G*′ ~100 Pa and ~1000 Pa. Traction stresses fluctuate over time. **(B)** Peak traction stress and **(C)** average traction stress as a function of substrate stiffness *G*′. Average traction stress values for a substrate stiffness of *G*′ ~ 10 kPa were excluded because most deformations were below our optical resolution limit. N: number of analyzed cells. ^**^p < 0.01; ^***^p < 0.001.

The traction stress (force per unit area) exerted by individual microglial cells, which is responsible for the observed substrate deformations, showed fluctuations over time (Figure 4A), indicating that force generation is a dynamic process. We did not find any distinct traction stress patterns as a function of substrate stiffness (Supplementary Figure 2). Peak as well as average traction stresses exerted by microglial cells changed significantly with substrate stiffness (*p* < 10^−12^, Kruskal-Wallis ANOVA and *p* < 10^−14^, One-Way ANOVA, respectively). Within the investigated range, traction forces increased with substrate stiffness (*p* < 0.01 for all comparisons, Mann-Whitney *U*-test) (Figures 4B,C).

#### Influence of LPS on traction stresses

In numerous pathological processes in the CNS, microglial cells become activated and migrate toward the stimulus. Lipopolysaccharide (LPS), as part of the outer membrane of gram-negative bacteria, elicits a strong immune reaction, which is in the CNS initiated by microglia (abd-el-Basset and Fedoroff, 1995; Nakamura, 2002). In order to observe the influence of microglia activation on the forces exerted by the cells, we performed traction force experiments on microglia treated with 1–2 μg/ml LPS.

**Figure 5:**
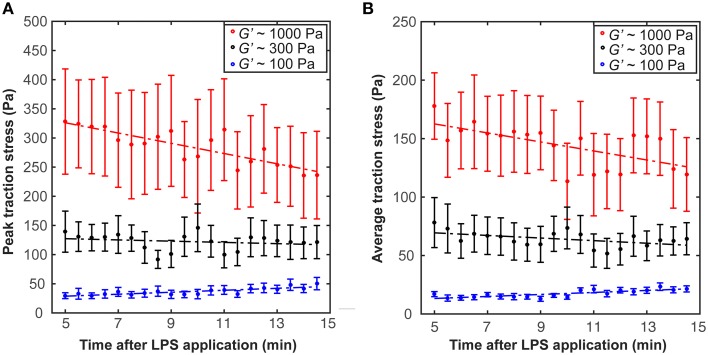
**Microglia activation through LPS changes traction stresses**. After 5 min incubation in LPS, **(A)** peak traction stresses and **(B)** average traction stresses decreased on stiffer substrates (*G*′ ~ 300 or 1000 Pa) but slightly increased on soft gels of *G*′ ~ 100 Pa (shown is the mean ± SEM).

After incubation in LPS-containing medium for ~5 min, we found a trend for traction forces to decrease within 10 min on stiffer substrates (1000 Pa and 300 Pa) and to increase slightly on 100 Pa gels (Figure 5). Testing if the slopes of the peak traction stresses over time significantly differed from 0 yielded a *p*-value of 0.06 at 1 kPa (one sample *t*-test); for all other conditions *p* > 0.06. Investigating the dependence of peak and average traction stresses on substrate stiffness *and* 10–15 min LPS treatment revealed interaction *p*-values of 0.002 and 0.004 (Two-Way ANOVA), respectively, indicating that the substrate stiffness-dependent traction forces significantly changed when microglia were activated by LPS.

### Durotaxis of microglia

CNS tissue is mechanically heterogeneous (Elkin et al., 2007; Christ et al., 2010; Franze et al., 2011; Iwashita et al., 2014; Koser et al., 2015). When microglial cells migrate through CNS tissue, they will thus encounter regions with different mechanical properties. To test if local changes in the stiffness of the environment impact microglia migration, we cultured microglial cells on compliant substrates incorporating stiffness gradients of ~8.35 Pa/μm and tracked the trajectories of actively migrating cells using time lapse microscopy (see Materials and Methods and Equation 1).

**Figure 6:**
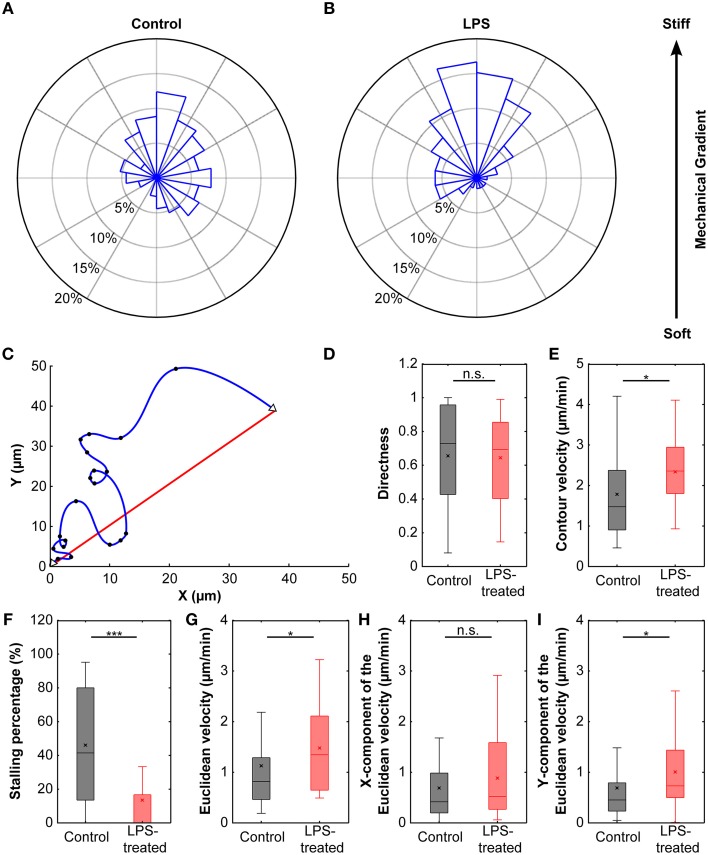
**Durotaxis of microglial cells**. **(A)** Distribution of microglial cell positions at the end of an experiment. Cells were cultured on compliant substrates (~5 kPa) incorporating a stiffness gradient of ~8 Pa/μm and preferentially migrated toward the stiffer side of the substrate (*N* = 38). **(B)** After activation with LPS, microglia durotaxis was significantly enhanced (*N* = 22). Rose plots were obtained from binned endpoints (bin size: of 60°, with 20° overlap). **(C)** Schematic plot of microglia migration. Start and end positions are marked with open triangles; black dots indicate cell positions recorded during time-lapse imaging. The blue curve shows the actual migration path (“contour”), the red line indicates the Euclidean distance between start and end position. **(D)** The directness D was similar for control and LPS-treated cells (*p* > 0.7, Mann-Whitney *U*-test). **(E,G)** Contour velocity *v*_*c*_ and Euclidean velocity *v*_*e*_ significantly increased after application of LPS (*p* < 0.05, Mann-Whitney *U*-test). **(F)** Stalling phases were highly reduced after LPS treatment (*p* < 10^−4^, Mann-Whitney *U*-test). **(H,I)** While the component of the Euclidean velocity perpendicular to the mechanical gradient (x-component) was similar for control and LPS-treated cells (*p* > 0.2, Mann-Whitney *U*-test), the component toward the stiffer side of the gradient (y-component) was significantly increased after application of LPS (*p* < 0.05, Mann-Whitney *U*-test). ^*^p < 0.05; ^***^p < 0.001.

Cells cultured on these compliant substrates migrated comparatively straight; the median directness *D* (Equation 2), which is the ratio between the shortest linear distance between the start and endpoints of a path and the total travel distance, was 0.73. Furthermore, microglia stopped frequently, with a median stopping rate of 41% (i.e., no movement between 41% of consecutive frames, see Materials and Methods), and traveled with a median contour velocity *v*_*c*_ (based on the contour length of the migration path; Equation 3) of 1.48 μm/min, and a median Euclidean velocity *v*_*e*_ (based on a straight line between start and end position; Equation 4 and Figure 6C) of 0.82 μm/min (Figure 6). Endpoints of the migration paths showed a non-uniform circular distribution (*p* < 0.01, Rayleigh test) and were strongly biased toward the stiffer side of the gradient (Figure 6A), indicating that microglial cells showed durotaxis.

#### LPS treatment changes microglia migration behavior

To test if the activation of microglia changed their tendency to migrate toward stiffer substrates, we then applied LPS to cells seeded on gels with stiffness gradients.

The directness of LPS-treated cells *D*_*LPS*_ = 0.70 was similar to that of control cells (*p* > 0.7, Mann-Whitney *U*-test). However, cells sped up by ~60% compared to untreated cells (median contour velocity of 2.36 μm/min compared to 1.48 μm/min; *p* < 0.05, Mann-Whitney *U*-test), which can be attributed to a significant decrease in median stall phases (0% and 41% for LPS-treated and control cells, respectively; *p* < 10^−4^, Mann-Whitney *U*-test). The median Euclidean velocity of LPS-treated cells was significantly higher as well (1.35 μm/min; *p* < 0.05, Mann-Whitney *U*-test) (Figures 6D–G).

Endpoints of migration were even stronger biased toward the stiffer side of the gradient (Figure 6B) with a highly non-uniform circular distribution (Rayleigh test of uniformity for endpoints; *p* < 0.001). When splitting the Euclidean velocity into its components parallel (*v*_*e,x*_) and perpendicular (*v*_*e,y*_) to the stiffness gradient, the velocity perpendicular to the gradient was similar in control and LPS-treated microglia (*v*_*e,x control*_ = 0.42 μm/min, *v*_*e,x LPS*_ = 0.52 μm/min; p > 0.2, Mann-Whitney *U*-test). However, the velocity toward the stiffer side of the gradient was significantly higher in LPS-treated cells (*v*_*e,y control*_ = 0.45 μm/min, *v*_*e,y LPS*_ = 0.73 μm/min; *p* < 0.05, Mann-Whitney *U*-test) (Figures 6H,I).

### Relating traction forces to durotactic behavior

#### Mathematical description of substrate stiffness-dependent traction forces

**Figure 7:**
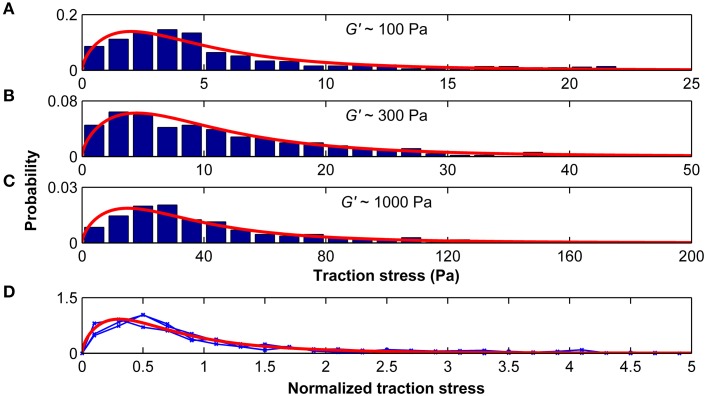
**Traction stresses are distributed according to a Burr distribution**. Stress distributions for microglia growing on substrates of *G*′ ~100, ~300, and ~1000 Pa are shown in **(A–C)**, respectively. Red curves represent the best three-parameter Burr distribution fits. **(D)** The stress distributions were normalized by their standard deviation. Blue curves show the normalized stress distributions for *G*′ ~100, ~300, and ~1000 Pa, which were statistically similar (*p* > 0.25, Kruskal-Wallis ANOVA). The red curve shows the Burr distribution fit for all data combined.

The distributions of average stresses we found on all substrates (except substrates of 10 kPa, which were excluded because most deformations were below our optical resolution limit) can be well described by a Burr type XII distribution (Figures 7A–C). The probability density function *p* of the three-parameter Burr distribution is

(9) $$p\left(\sigma ;\alpha ,c,k\right)=\frac{kc{\left(\frac{\sigma }{\alpha }\right)}^{c-1}}{\alpha {\left[1+{\left(\frac{\sigma }{\alpha }\right)}^{c}\right]}^{k+1}},$$

with the positive scale parameter α and the two positive shape parameters *c* and *k*. When the stress distributions were normalized by their standard deviations, they were statistically similar (*p* > 0.25, Kruskal-Wallis ANOVA) and fell on one master curve (Figure 7D). Therefore, only the scale parameter α but not the shape parameters *c* and *k* depended on the substrate stiffness.

The ratio between peak stress and standard deviation of the stress distribution was similar for the different substrates (σ_max_/SD(σ) = 4.48±0.01; *p* > 0.1, One-Way ANOVA), suggesting that the peak stress alone is sufficient to predict the distribution of microglial traction stresses on compliant substrates of arbitrary stiffness.

**Figure 8:**
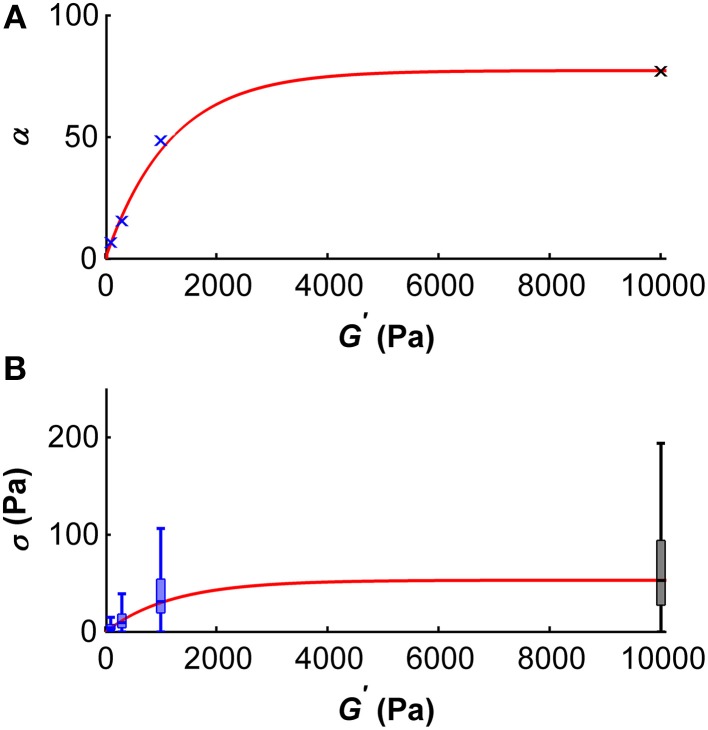
**Dependency of the scale parameter α (A) and the traction stress σ (B) on the gel's shear modulus *G*′**. **(A)** Blue crosses represent α values determined for *G*′ ~100, ~300, and ~1000 Pa, the black cross represents an α value approximated for *G*′ ~ 10 kPa using the master curve and the constant peak stress to standard deviation ratio. An initial regime of fast increase of α (which scales proportionally with the median traction stress) was observed for shear moduli below ~2 kPa. The red curve represents the best fit with an adjusted *R*^2^-value of 0.99. **(B)** Boxplots of the traction stresses σ for *G*′ ~100, ~300, and ~1000 Pa are shown in blue. Black boxplot shows the estimated traction stress σ for *G*′ ~10 kPa. An initial regime of fast increase of σ was observed for shear moduli below ~2 kPa. The red curve represents the best fit through the median traction stresses with an adjusted *R*^2^ value of 0.99.

Using these results, we can make a prediction on the dependency of the scaling parameter α on the shear modulus *G*′ of the substrate microglial cells grow on (Figure 8A). This dependency can be described by

(10a) $$\alpha \left(G^{\prime }\right)=-ae^{-bG^{\prime }}+a,$$

with the fitting parameters *a* = 77.28 and *b* = 0.00086/Pa. The adjusted *R*^2^–value of this fit is 0.99. In the same way, the dependency of the median traction stress on the shear modulus can be described by

(10b) $$\sigma \left(G^{\prime }\right)=-ae^{-bG^{\prime }}+a,$$

with the fitting parameters *a* = 53.2 Pa and *b* = 0.00083/Pa (Figure 8B). The adjusted *R*^2^–value of this fit is 0.99.

#### A random walk-based description of microglia durotaxis

To investigate why microglial cells preferentially migrated toward stiffer substrates, we first tested if there was a dependency of the movement direction of a cell on its previous direction (governed by the turning angle φ; Equations 5a,b). There was no difference in the likelihood of moving forward (0.22 ± 0.04), backward (0.23 ± 0.03), left (0.25 ± 0.04) or right (0.30 ± 0.04; *p* > 0.4, Kruskal-Wallis ANOVA). A similar trend was observed after exposing microglia to LPS (*p* > 0.95). The correlation index *CI* ∈ [0,1] (Equation 7b), which is a measure of the correlation between the angle of two consecutive steps, was 0.04 in both conditions, indicating that turning angles were not correlated, and that the observed durotaxis cannot be explained by a persistency in motion with non-uniform starting conditions.

Next, we investigated the dependence of a cell's motion on the gradient direction (governed by the direction angle θ; Equations 6a,b). The likelihood of a cell to move along the gradient (0.50 ± 0.04; 0.48 ± 0.05 for LPS-treated cells) was the same as perpendicular to it (0.50 ± 0.04; 0.52 ± 0.05 for LPS-treated cells) (*p* > 0.5 for both conditions). However, cells preferentially moved toward the stiffer side of the gradient (0.31 ± 0.04; 0.32 ± 0.05 for LPS- treated cells) compared to the softer side (0.19 ± 0.02; 0.16 ± 0.03 for LPS-treated cells) (*p* < 0.01 in both conditions). The bias index *BI* ∈ [0,1] (Equation 8), which is a measure of the correlation between the step direction (i.e., angle) and the stiffness gradient, was 0.15 in control cells and 0.16 in LPS-treated cells, indicating that cell migration was biased along the mechanical gradient. Microglia activation through LPS-treatment did not alter the impact of the stiffness gradient on the migration direction (*p* > 0.5, Mann-Whitney *U*-test).

As not only the likelihood to move in a certain direction can influence the direction bias of cells but also a dependence of the step-length (and thus velocity) on directionality, we studied step-length distributions of microglia migrating on stiffness gradients. While step-lengths (measured every minute) were independent of the average gradient direction (*d*_∥*G*_ ≈ *d*_⊥*G*_ = 1.35 μm; 1.27 μm for LPS-treated cells; *p* > 0.4 for both conditions, Mann-Whitney *U*-test), they did depend on the direction of the previous step. Lengths of steps parallel to the previous movement direction *d*_∥*M*_ = 1.41 μm (1.30 μm for LPS-treated cells) were larger than step lengths perpendicular to it *d*_⊥*M*_ = 1.30 μm (0.84 μm for LPS-treated cells) (*p* < 0.05, *p*_*LPS*_ = 0.078, Mann Whitney *U*-test). Furthermore, we found a tendency of the step length to be higher parallel *d*_∥*M*1_ = 1.76 μm (1.47 μm for LPS-treated cells) than antiparallel *d*_∥*M*2_ = 1.32 μm (1.27 μm for LPS-treated cells) to the previous movement direction (*p* = 0.089, *p*_LPS_ = 0.068, Mann Whitney *U*-test). These results combined indicated that cells migrated faster if they did not change their direction.

**Figure 9:**
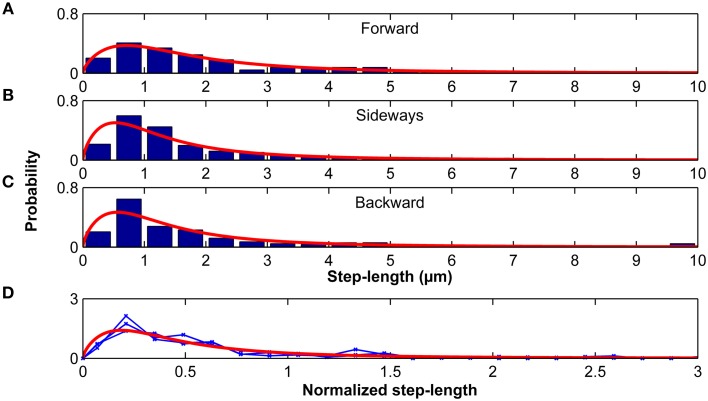
**Step lengths are distributed according to a Burr distribution**. Step-length distributions for the forward **(A)**, sideways **(B)** and backward movements **(C)** of microglia relative to their previous step direction. Red curves represent the best Burr distribution fits. **(D)** Step-length distributions were normalized by their standard deviation. Blue curves show the normalized stress distributions for the forward, sideways, and backward movements, which were statistically similar (*p* > 0.20, Kruskal-Wallis ANOVA). The red curve shows the Burr distribution fit for all data combined.

Step-length probability distributions, which depended on the direction of the previous step, were well-approximated by the Burr distribution (Figure 9; Equation 9). When step-length distributions were normalized by their standard deviations, they were statistically similar (*p* > 0.20, Kruskal-Wallis ANOVA) and fell again on one master curve (Figure 9D). Therefore, only the scale parameter α but not the shape parameters *c* and *k* depended on the turning angle φ.

Next, we tested if we can predict the directional bias of microglia migrating on stiffness gradients (e.g., endpoints position, direction angle distribution of endpoints, directness, and the velocity) using a 2D biased random walk model with step-length distributions dependent on the turning angle φ. The walk was modeled by moving 228 cells (6 times as many as in the experiments) at each time step (corresponding to 1 min) by a length *l* in a direction angle θ at a time distribution matching that of the experimental data. The bias was imposed on the system by setting the direction angle distribution of θ to be a circular normal (or von Mises) distribution of the form

(11) $$p\left(\theta ;\kappa \right)=\frac{e^{\kappa \cos\theta }}{2\pi I_{0}(\kappa )},$$

where *I*_0_*(*κ*)* is the modified Bessel function of order 0. The parameter κ was set to 0.30, accounting for the ~31% likelihood to move to the stiffer side of the gradient, the ~19% likelihood to move to the softer side of the gradient, and the ~50% likelihood to move perpendicular to the gradient, as found in our experiments. The step-length *l* was set to 0 μm with a 41% chance to account for stall phases. Otherwise, the step-length *l* was randomly selected from a Burr distribution (see Equation 9); a random number *R* ∈ [0,1] was transformed by the inverse cumulative distribution function of the Burr distribution

(12) $$l\left(R;\alpha ,c,k\right)=\alpha {\left[\frac{1}{{\left(1-R\right)}^{1/k}}-1\right]}^{1/c}.$$

**Figure 10:**
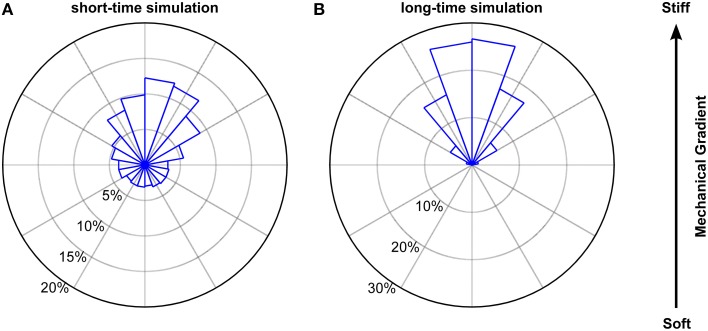
**Simulated biased random walk of microglial cells migrating on a mechanical gradient**. **(A)** Simulation for 228 cells (6 times as many as in our experiments) with a migration time distribution matching that of the experiments. Microglia were similarly biased toward the stiffer side of the substrate as found in the experiments (*p* > 0.1, Kuiper's test). **(B)** Simulation for 1000 cells migrating 1000 min. On longer time scales, cells showed a robust migration toward the stiffer side of the gradient (*p* < 0.01, Kuiper's test).

To account for the dependence of the step length on the turning angle φ, we applied the φ-dependent fitting parameters of the Burr distributions used in Figure 9. The results of our simulation closely matched our experimental findings. Endpoints were strongly biased toward the stiffer side of the gradient (Figure 10), with a non-uniform circular distribution (*p* < 10^−12^, Rayleigh test). Furthermore, both the angular distribution of endpoints (*p* > 0.1, Kuiper's test) and their coordinates were similar to our experimental data (*p* > 0.05 and *p* > 0.85 for the coordinates perpendicular and along the gradient, respectively, Mann Whitney *U*-test). While the contour velocity was similar (1.30 μm/min, *p* > 0.10), the directness *D* was lower compared to the experimental data (0.44; *p* < 10^−3^). Accordingly, also the Euclidean velocity was significantly reduced (0.47 μm/min, *p* < 10^−3^). However, when we adapted the number of simulated cells to that of the experiments (*N* = 38) and matched the stalling percentage distributions, the directness was similar (0.55; *p* > 0.25). Hence, a biased random walk model approximates our experimental findings very well.

Next, we simulated the long time behavior of microglia (1000 cells for 1000 min). As expected, endpoint positions of simulated cells were biased even stronger toward the stiffer side of the gradient (*p* < 0.001 for the comparison with our experimental data, Kuiper's test) (Figure 10B) with a clearer non-uniform circular distribution (*p*≈0, Rayleigh test of uniformity for endpoints). Furthermore, the median contour velocity did not change compared to our experimental data (1.40 μm/min vs. 1.48 μm/min in the experiments; *p* > 0.50, Mann-Whitney *U*-test). The component of the Euclidean velocity toward the stiffer side of the mechanical gradient, which is an expression for the migration velocity along the gradient for long time periods, was 0.215 ± 0.002 μm/min (compared to 0.45 μm/min in the experiments), while the component perpendicular to the gradient was close to 0 μm/min. The median directness *D* was highly reduced (0.16; *p* < 10^−18^).

#### Connecting traction forces and durotaxis through a stress fluctuation model

We finally used a simple numerical simulation to test if the observed bias of migrating microglia toward the stiffer side of their substrate may be caused by the interplay between the traction stresses transmitted to the substrate, the local substrate stiffness underneath the cell, and the steepness of the stiffness gradient. For this purpose, we idealized the cell's interface with the hydrogel to be a perfect circle, which we discretized into pixels of 0.3 × 0.3 μm^2^. We made the following assumptions: (i) The cell is directly tethered to the substrate in each pixel, and it exerts shear stresses on each pixel which are distributed according to the master curve of the stress distributions (Figure 7D). (ii) All stresses are radially and inwardly orientated. (iii) At each pixel, the substrate can move individually and has a constant shear storage modulus *G*′. (iv) Cells move by attaching to the surface, contracting and reattaching at the new position.

The directional bias of cell movement on substrates with changing mechanical properties can be illustrated by simplifying the problem to a one-dimensional cell consisting of two sides (or one-dimensional “pixels”) exerting forces on a linearly elastic half-space. Let's assume that the one-dimensional shear stress on one side is τ_1_ and on the second side τ_2_ = −τ_1_. The shear strain γ on each side of the cell is then calculated by

(13a) $$\gamma =\frac{\tau }{G^{\prime }},$$

where *G*′ is the shear storage modulus of the substrate underneath that pixel. The shear strain γ is linked to the surface displacement Δ*l* by

(13b) $$\gamma \approx \frac{△l}{L},$$

where *L* is the effective thickness of the gel (up to which the substrate is deformed), which we assume to be constant. Thus, the one-dimensional deformation underneath both cell sides is

(13c) $$△l\approx L\frac{\tau }{G^{\prime }}.$$

A stiffness gradient in the substrate will thus impose a migratory bias on the cell, leading to a directed migration of length Δ*L*, calculated by

(13d) $$\begin{array}{l} △L=-\frac{△l_{1}+△l_{2}}{2}\approx -\frac{L}{2}\left(\frac{\tau_{1}}{G_{1}^{{}^{\prime }}}+\frac{\tau_{2}}{G_{2}^{{}^{\prime }}}\right), \end{array}$$

where Δ*L* ≠ 0 μm only if $G_{1}^{{}^{\prime }}\neq G_{2}^{{}^{\prime }}$. Hence, it is the difference in shear modulus of the substrate underneath the cells that causes the bias in microglia migration. This argumentation can be easily extended to three dimensions according to

(14) $$\vec{△L}\approx -\varepsilon \sum_{i=1}^{N}\frac{1}{G_{i}^{{}^{\prime }}}\vec{\tau_{i}},$$

where *N* is the number of pixels underneath the cell, and ε = *L*/*N.* For the simulation, we used a gradient steepness of 8.35 Pa/μm, and positioned the middle of the cell at a shear modulus of *G*′ ~ 5 kPa.

Simulating, 100 cells moving 50 steps each led to a von Mises distribution of the direction angle θ with *k* = 0.29, which is very close to what we assumed in our random walk model (see Equation 11). We found no difference in the likelihood of moving forward (0.24 ± 0.01), backward (0.26 ± 0.01), left (0.25 ± 0.01) or right (0.25 ± 0.01) relative to the previous movement direction (p > 0.60, Kruskal-Wallis ANOVA). However, the probability of moving toward the stiffer side of the gradient was significantly higher (0.31 ± 0.01) than to the softer side (0.20 ± 0.01) (*p* < 10^−21^, Mann-Whitney *U*-test). These results were very similar to our experimental data (*p* > 0.3, Mann-Whitney *U*-tests). Furthermore, the simulated step lengths were distributed according to a Burr distribution, as in our experiments and as used in our random walk model. Thus, our stress fluctuation model nicely captures the main features of the movement bias of microglia along mechanical gradients.

## Discussion

In this study, we found that microglial cells adapt their morphology and cytoskeleton to the stiffness of their environment. Their spread area increased on stiffer substrates, similarly as that of astrocytes (Georges et al., 2006; Moshayedi et al., 2010) (Figure 1). Furthermore, their morphology increased in complexity, in agreement with a previous report (Moshayedi et al., 2014): microglia morphologies changed from round and weakly polarized with numerous filopodia-like protrusions on soft to a lamellipodia-dominated, strongly polarized phenotype with many large cell processes on stiffer substrates (Figure 2) (Bergert et al., 2012; Liu et al., 2015). Actin was found mostly in the periphery on soft substrates, while it was distributed throughout the cells on stiffer substrates. These differences in cytoskeletal arrangements could have substantial impact on microglia, as the dynamic state of the actin cytoskeleton profoundly affects their behavior and function (Uhlemann et al., 2015).

Traction forces exerted by microglia initially increased with substrate stiffness and saturated around a shear modulus of ~5 kPa (Figures 4, 8B). Similar trends have been described for other cell types (Ghibaudo et al., 2008; Califano and Reinhart-King, 2010; Han et al., 2012; Koch et al., 2012; Trichet et al., 2012) and were predicted using active matter theory (Marcq et al., 2011), suggesting this to be a general tissue cell behavior. However, the specific substrate stiffness at which traction stresses saturate is cell type-specific (Ghibaudo et al., 2008; Califano and Reinhart-King, 2010; Han et al., 2012; Koch et al., 2012; Trichet et al., 2012).

Stress distributions of microglia are well-described by a Burr distribution (Figure 7). The shape parameters *c* and *k* are independent of the surrounding stiffness, while the scale parameter α depends on the shear modulus of the substrate. At substrates of *G*′ ~ 5 kPa, α reaches a plateau, and estimated median microglial traction forces saturate (Figure 8).

Microglial cells were most sensitive to substrate rigidity in a range of *G*′ between 0 and ~2000 Pa (Figure 8), which matches the range of CNS tissue stiffness (Franze et al., 2013). Thus, microglia seem to be mechanically tuned to their natural environment. Furthermore, they are significantly stronger than their neighboring CNS neurons; the mean traction stresses exerted by hippocampal neurons are an order of magnitude lower than microglial traction stresses measured in this study (Koch et al., 2012).

Most microglia grown on compliant cell culture substrates were not migratory, as usually found for microglia grown on glass or tissue culture plastics (abd-el-Basset and Fedoroff, 1995; Lively and Schlichter, 2013). Nevertheless, when exposed to stiffness gradients in the substrate, a subset of microglial cells migrated—preferentially toward the stiffer side in a process called durotaxis (Lo et al., 2000; Isenberg et al., 2009; Kuo et al., 2012; Plotnikov et al., 2012; Raab et al., 2012; Vincent et al., 2013) (Figure 6). The migratory bias imposed by the mechanical gradient was similarly strong as seen in chemotaxis of leucocytes toward LTB4 (Foxman et al., 1999), suggesting a similar importance of mechanical signaling in immune cell attraction as chemical signaling.

Microglia migration on stiffness gradients was more likely to be in the direction of increased stiffness. Furthermore, step lengths were larger when moving parallel than perpendicular to the previous step, effectively amplifying migration toward stiff (as the likelihood of a step toward the stiffer side was higher). Microglia durotaxis was well-approximated using a 2D biased random walk model with changing step-lengths (distributed according to a Burr distribution, Figure 9) depending on the turning angle φ.

Differences in the local stiffness of the substrate underneath the cells were sufficient to explain durotaxis by a purely physical mechanism as previously discussed (Bischofs and Schwarz, 2003; Lazopoulos and Stamenovic, 2008), and as predicted by our stress fluctuation model (Figure 10). Thus, microglia showed durotaxis even when they were migrating on substrates whose stiffness was beyond their sensitive range, i.e., *G*′ > 2 kPa. On these substrates, cellular traction forces can be considered rather constant across an individual cell, indicating that durotaxis does not require an intracellular traction force gradient.

Instead, a cell pulling on the substrate underneath will deform softer substrates more than stiffer ones, and a stiffer substrate will provide more traction. If a cell pulls on a substrate incorporating a stiffness gradient, it will thus pull itself toward the stiffer side. On softer substrates below *G*′ ~ 2 kPa, microglia will pull significantly stronger on the stiffer side (Figures 4, 8), modulating the durotactic effect. However, even if the local stresses on both sides of the cell are similar (i.e., on substrates with *G*′ > ~5 kPa as in our experiments), cells will show the same durotactic bias as long as the ratio of the steepness of the gradient to the absolute substrate stiffness remains constant. This finding adds to previous work showing that the gradient strength is important for cellular durotaxis (Isenberg et al., 2009; Vincent et al., 2013), and it fits to the idea that a cell can only measure the stiffness of the surrounding relative to its own stiffness (Schwarz et al., 2006; Trichet et al., 2012).

In addition to this physical mechanism, cell type-specific mechanotransduction signaling pathways (Franze et al., 2009; Plotnikov et al., 2012; Raab et al., 2012), and stiffness-dependent local changes in cell morphology, such as stress fiber formation (Trichet et al., 2012), cell polarization (Isenberg et al., 2009; Raab et al., 2012), focal adhesion formation (Plotnikov et al., 2012), or a local change in their contact angle at the substrate surface (Style et al., 2013), could contribute to the observed durotactic behavior. Furthermore, microglia also respond to chemical guidance molecules. If exposed to mechanical and chemical cues, cells will integrate both types of signals and respond accordingly. Chemotaxis can be implemented in our stress fluctuation model in the future by adding an appropriate term.

Nowadays, neural implants (e.g., electrodes) are routinely used to treat patients suffering from Parkinson's disease and clinical depression. A major problem with long-term implants is their encapsulation by local immune cells in a process termed foreign body reaction (He and Bellamkonda, 2008; Tresco and Winslow, 2011). In the brain, microglial cells are activated by an implant and migrate toward it, which significantly contributes to the progression of foreign body reactions. Our stress fluctuation model provides a useful starting point to understand why and how microglia migrate toward stiff implants in the CNS.

At least three factors are likely to contribute to a stiffening of the tissue surrounding neural implants, and thus to establishing a local stiffness gradient. (i) Contact of microglial cells with stiff materials leads to their activation (Moshayedi et al., 2014). Glial cell activation, on the other hand, which is correlated with an increased expression of intermediate filaments, leads to a stiffening of the cells (Lu et al., 2011). (ii) The increase in traction forces we observed on stiffer substrates (Figure 4) is likely to lead to an increase in cytoskeletal tension, contributing to a stiffening of cells on stiffer substrates (Engler et al., 2004; Solon et al., 2007; Zemel et al., 2010). (iii) Both microglia migration toward the implant and their proliferation at the implant lead to a local increase in cell density, which again contributes to a local stiffening of the tissue (Koser et al., 2015). Thus, tissue surrounding neural implants is likely to be stiffer than healthy tissue further away. For the same reasons, microglia in contact with this stiffer tissue probably also stiffen to some degree. Thus, the three-dimensional arrangement of microglia around neural implants should effectively lead to a stiffness gradient in the tissue, attracting further glial cells to the implant in a durotactic fashion, and thus facilitating foreign body reactions.

Immuno-activation by LPS-treatment significantly changed microglial traction stresses. Forces exerted by cells grown on stiffer substrates (*G*′ ~ 300 or 1000 Pa) decreased after LPS-activation, while forces slightly increased when cells were cultured on soft substrates of *G*′ ~ 100 Pa (Figure 5). At the same time, LPS-application led to a significant increase in migration velocity and a decrease in stalling phases (Figure 6). Faster migration (and lower traction forces) are often a consequence of decreased adhesion (Fournier et al., 2010). LPS treatment furthermore focused microglia migration toward the stiffer side of the substrate, likely because of the strong decrease in stalling phases, which should lead to an enhancement of the durotactic bias. The activation-dependent tuning of the mechanical interactions between microglia and their environment might thus be an important mechanism controlling microglia morphology and function *in vivo*.

Ultimately, durotaxis of microglia might be required during normal CNS functioning and to react to a multitude of CNS disorders. Stiffness gradients in neural tissue at the cellular scale exist throughout life (Elkin et al., 2007; Christ et al., 2010; Franze et al., 2011; Iwashita et al., 2014; Koser et al., 2015). Furthermore, many CNS pathologies are accompanied by changes in neural tissue stiffness (Murphy et al., 2011; Riek et al., 2012; Schregel et al., 2012; Streitberger et al., 2012; Chauvet et al., 2015), which are potentially sensed by microglia. Local gradients in tissue stiffness might provide an important signal contributing to microglia activation and migration toward the pathological stimulus. On the other hand, microglia durotaxis might also contribute to the pathogenesis of CNS disorders, and to facilitating foreign body reactions to neural implants (Moshayedi et al., 2014). Future, studies will shed more light on how cell mechanics is involved in physiological and pathological processes in the CNS.

## Author contributions

KF conceived the project; LB, DK, GS, MG and KF designed the research; LB, DK, HG, RS, and EU performed research; LB, DK, and KF analyzed data; LB, DK, and KF wrote the paper, with contributions from all co-authors.

### Conflict of interest statement

The authors declare that the research was conducted in the absence of any commercial or financial relationships that could be construed as a potential conflict of interest.
