# Supplementary material for: Microglia mechanics: immune activation alters traction forces and durotaxis
Source: Front Cell Neurosci. 2015 Sep 23;9:363. doi: 10.3389/fncel.2015.00363 (PMC4585148; doi:10.3389/fncel.2015.00363)
Supplement: Supplementary file 1 [file DataSheet1.PDF]

Supplementary Material

Microglia mechanics: immune activation alters traction forces and durotaxis

Lars Bollmann‡, David E. Koser‡, Rajesh Shahapure, Hélène O. B. Gautier, Gerhard A. Holzapfel, Giuliano Scarcelli, Malte Gather, Elke Ulbricht, Kristian Franze*

‡ These authors contributed equally to this work.

*** Correspondence:** kf284@cam.ac.uk

1 Supplementary Figures

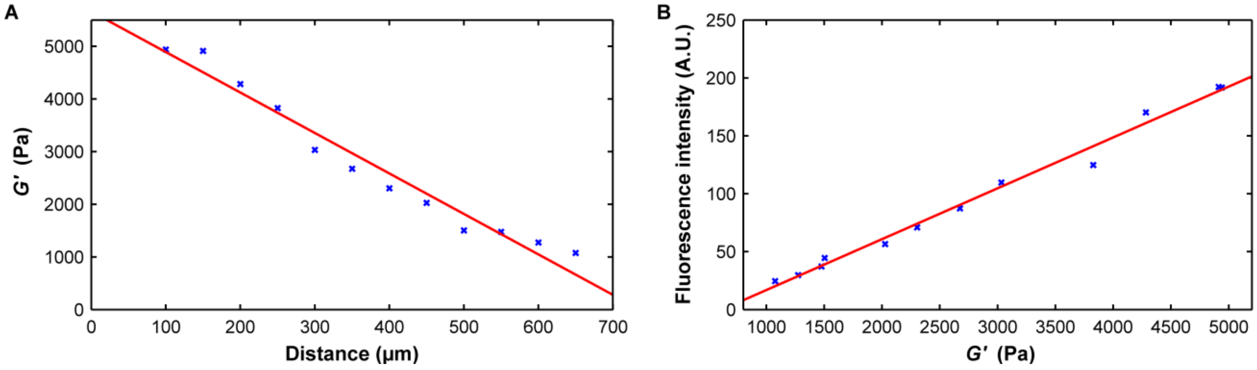

Supplementary Figure 1. Characterization of the stiffness gradient substrates. (A) Atomic force microscopy indentation experiments revealed a linear relationship between the shear modulus G' (assuming a Poisson's ratio $\nu = 0.5$) and the position along the substrate (Pearson's correlation coefficient $\rho = -0.98$). (B) The stiffness gradient strongly correlated with a gradient in fluorescence intensity (Pearson's correlation coefficient: $\rho = 0.99$). Therefore, fluorescence intensity can be used as a readout for the local shear modulus.

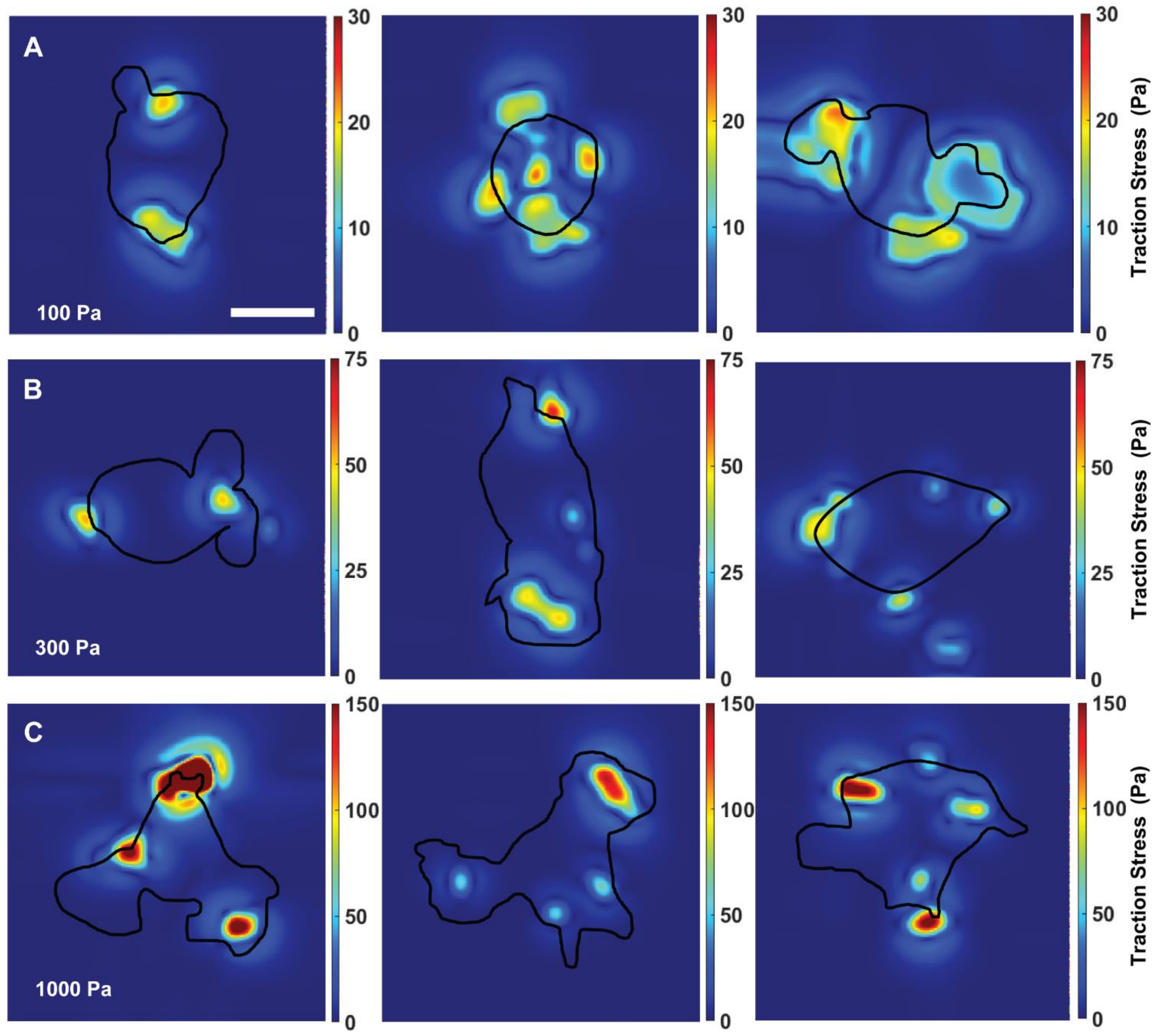

Supplementary Figure 2. Comparison of traction stress distributions in microglia cultured on compliant substrates with shear moduli of (A) 100 Pa, (B) 300 Pa, and (C) 1000 Pa. Black lines indicate the outlines of the cells. Traction stress fields varied significantly for different cells and over time, irrespective of substrate stiffness; we did not observe any distinct traction stress patterns as a function of substrate stiffness. Scale bar: 10 µm.
